# Pelvic skeletal metastasis of hepatocellular carcinoma with sarcomatous change: a case report

**DOI:** 10.1186/1746-1596-5-33

**Published:** 2010-05-25

**Authors:** Chih-Yu Chen, Yong-Te Hsueh, Tsung-Yu Lan, Wei-Hsin Lin, Karl Wu, Rong-Sen Yang

**Affiliations:** 1 Division of Orthopedics, Surgical Department, Far Eastern Memorial Hospital, Taipei, Taiwan; 2 Department of Orthopedic Surgery, National Taiwan University & Hospital, Taipei, Taiwan; 3 Department of Orthopedic Surgery, Taipei Medical University, Shuang Ho Hospital, Taipei, Taiwan

## Abstract

Sarcomatoid hepatocellular carcinoma (HCC) is a very rare histologic variant of HCC. The characteristic of skeletal metastatic sarcomatoid hepatocellular carcinoma has never been reported. We reported a patient with sarcomatoid hepatocellular carcinoma pelvic metastasis who presented with huge pelvic metastasis that had relatively small osteolytic lesion centrally located accompanied by huge bipeduncular invasive expansile lesions into surrounding soft tissue. The lesion showed almost non-isotope uptake in ^99m^Tc-methylene diphosphonate bone scintigraphy study. He underwent radiotherapy and tumor excision but the tumor rapidly recurred. In addition, serum α-fetoprotein level was never elevated beyond normal limit (< 20 ng/mL) through the whole course of treatment. We considered sarcomatoid hepatocellular carcinoma bone metastasis a highly aggressive lesion with unusual metastatic pattern. Surgical treatment with adequate safe margin in such a huge tumor with hypervascularity and extensive invasion in the pelvis was difficult; and radiotherapy maybe refractory regarding the sarcomatous nature. Therefore, debulking operation with local symptoms control may provide a better quality of life. And the clinical course suggests sarcomatoid hepatocellular carcinoma is derived from the transition of an ordinary hepatocellular carcinoma.

## Introduction

Sarcomatoid hepatocellular carcinoma (HCC) is a very rare histologic variant of HCC with an incidence of 1.8% in surgically resected cases and 3.9% to 9.4% in autopsied cases [1-4]. Although sarcomatous transformation of HCC had been discussed in a few reports [3,5]; it was never been reported in patients with bone metastasis.

The skeletal metastasis of sarcomatoid HCC have specific features of expansile, destructive nature, accompanied by large, bulky soft tissue masses; and these lesions often showed non-isotope uptake in ^99m^Tc-methylene diphosphonate (MDP) bone scintigraphy (BS) study [4,6-8].

We report a patient with sarcomatoid HCC pelvic metastasis. The patient presented with huge pelvic metastasis that had relatively small osteolytic lesion centrally located accompanied by huge bipeduncular invasive expansile lesions into surrounding soft tissue. He underwent radiotherapy and tumor excision. Unfortunately, the tumor rapidly recurred in short period. On the other hand, serum α-fetoprotein (AFP) level was never elevated beyond normal limit (< 20 ng/mL) after diagnosis of skeletal metastasis.

## Case Report

The patient was a 61 year-old male, who was a hepatitis B virus (HBV) carrier. He had hepatoma since November 2006 and underwent right hepatectomy. Pathologic analysis showed typical grade II to III HCC. Serum AFP level was greater than 3,000 ng/mL at that time. Abdominal sonography in July 2008 did not detect any viable liver tumor. However, hepatic tumor recurrence was noted since November 2008 and was treated 3 times with transarterial embolization (TAE) therapy from November 2008 to July 2009.

**Figure 1:**
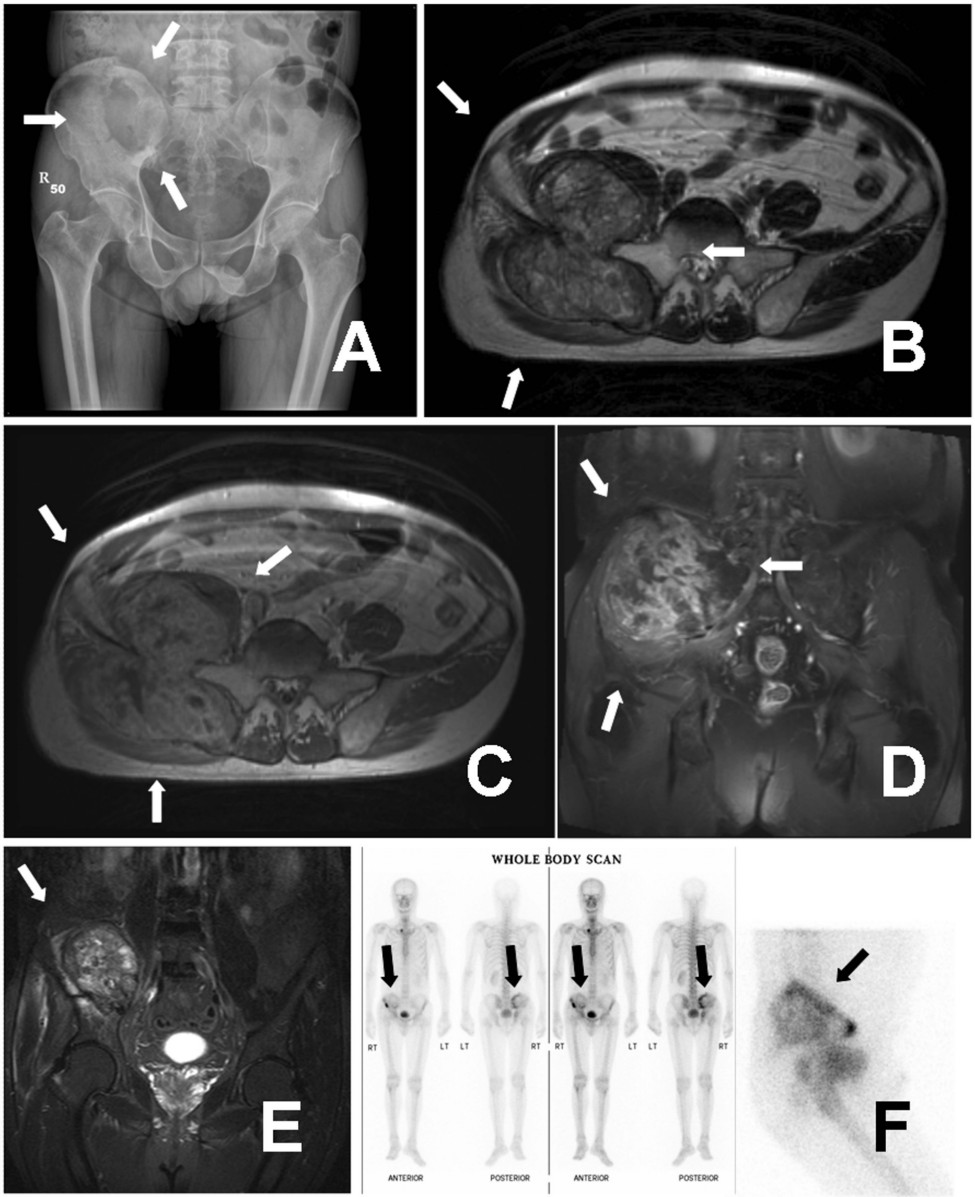
**Pelvis radiograph showed a large osteolytic lesion over right iliac bone near sacroiliac junction (white arrows) (A)**. MRI revealed a 9.6 × 9.4 × 9.0 cm bilobular huge tumor mass arising from right iliac wing with bony destruction and significant soft tissue extension (white arrows) (B to E). ^99m^Tc-MDP BS showed mildly hot areas at right iliac bone with a large, round cold area (black arrows) (F). B: axial T1-weighted image with contrast enhancement. C: axial T2-weighted image. D: coronal fat-suppressed (FS) T2-weighted image with contrast enhancement. E: coronal short tau inversion recovery (STIR) images.

On the other hand, a solitary right pelvic HCC metastasis was noted since July 2008; and was treated with palliative radiotherapy by a total dose of 5,250 cGy in 21 fractions from August 2008 to March 2009. The discomfort subsided temporarily after radiotherapy treatment. Unfortunately, the patient had progressive right low back pain with radiation to right lower extremity accompanied with right buttock knocking pain since 2009 April, and the symptom deteriorated in the following few weeks. Pelvis radiograph showed a large osteolytic lesion by right iliac bone near sacroiliac junction (Fig 1-A; white arrows). Magnetic resonance images (MRI) study revealed a 9.6 × 9.4 × 9.0 cm bilobular lesion arising from right iliac bone with heterogeneity and good enhancement after contrast (Fig 1-B to 1-E, white arrows). Bony destruction into adjacent sacrum and significant soft tissue extension were also noted, causing adjacent psoas, iliacus and gluteus muscles hyperemia. Besides, tumor compression of the right sciatic nerve was also suspected. ^99m^Tc-MDP BS showed mildly hot areas at right iliac bone with a large, round cold area (Fig 1-F; black arrows).

**Figure 2:**
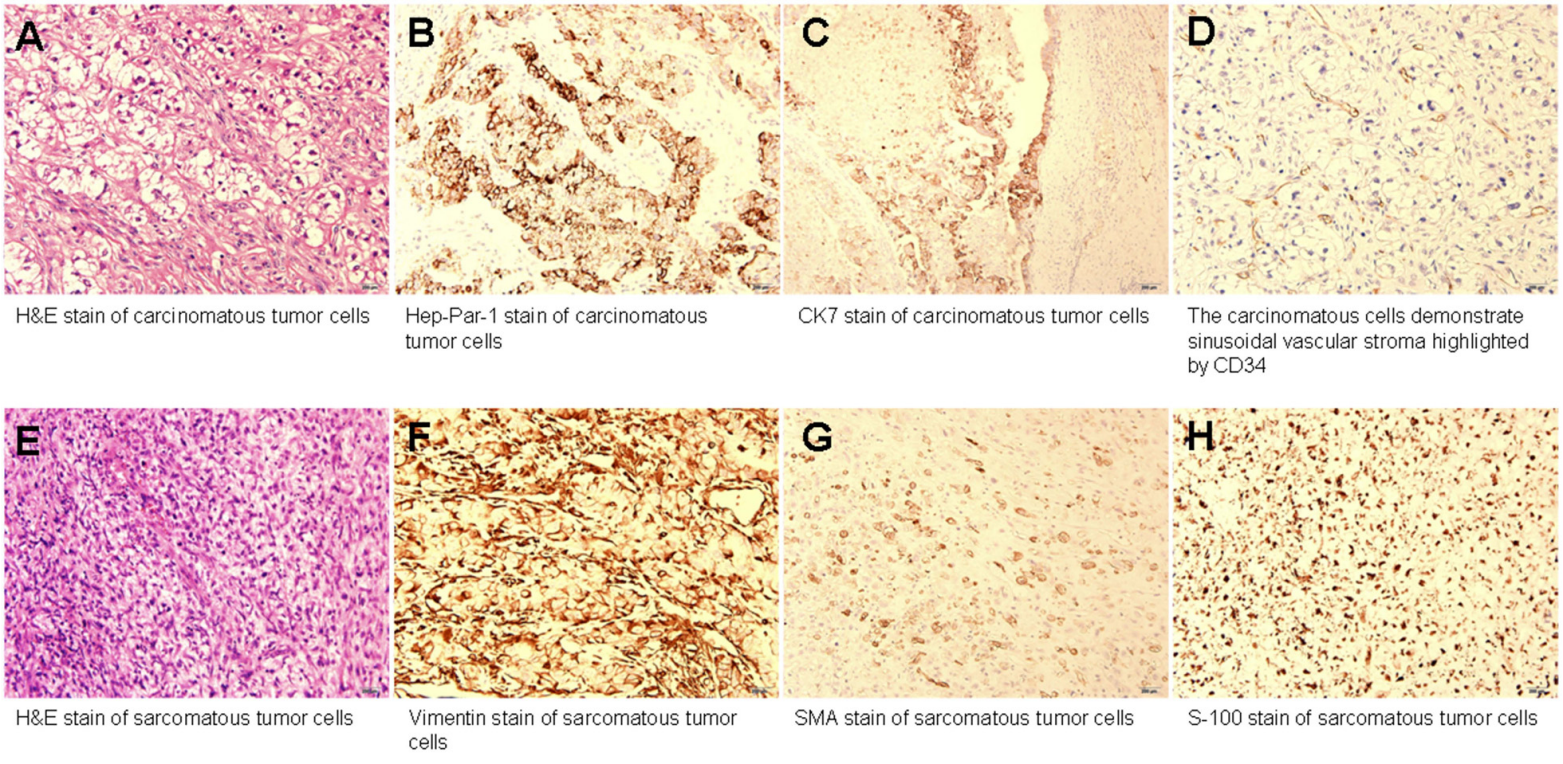
**Histopathologic pictures of the metastatic HCC**. Hematoxylin and Eosin (H&E) staining showed tumor cells infiltrate in the bony trabeculae and soft tissue (A and E). There were two patterns of tumor cells (A and E). Histopathologic pictures of the metastatic HCC. Hematoxylin and Eosin (H&E) staining showed tumor cells infiltrate in the bony trabeculae and soft tissue (A and E). There were two patterns of tumor cells (A and E). The carcinomatous tumor cells with pleomorphic nuclei and abundant cytoplasm arranged in solid sheets and nests were positive for HepPar-1 and cytokeratin-7 (CK7) staining (B and C). The carcinomatous tumor cells also demonstrate sinusoidal vascular stroma highlighted by CD34 (D). The other sarcomatous cells with little cytoplasm arranged in the myxo-chondroid background were positive for vimentin, SMA, and S-100 protein staining (F to H). These features are compatible with a metastatic hepatocellular carcinoma with sarcomatous change. Magnification: 200×.

The patient underwent tumor excision on June 8^th^, 2009. A hypervascular round mass about 10 cm in diameter located at right iliac wing was noted intraoperatively. The tumor had a relatively small central bony destruction about 2.5 × 2.0 cm in size and expanded into both inner and outer sides of the pelvic wall. These findings suggested that the whole tumor mass originated from a central bone metastasis and then gradually enlarged and invaded into soft tissue in both directions. The histopathological examination with Hematoxylin and Eosin (H&E) staining showed tumor cells infiltrate in the bony trabeculae and soft tissue. There were two patterns of tumor cells (Fig 2A and 2-E). The carcinomatous tumor cells with pleomorphic nuclei and abundant cytoplasm arranged in solid sheets and nests were positive for hepatocyte paraffin-1 (HepPar-1) staining (Fig 2-B) and cytokeratin-7 (CK7) staining (Fig 2-C). The carcinomatous tumor cells also demonstrated sinusoidal vascular stroma highlighted by CD34 (Fig 2-D). The other sarcomatous cells with little cytoplasm arranged in the myxo-chondroid background were positive for vimentin, smooth muscle actin (SMA), and S-100 protein staining (Fig 2-F to 2-H). These features were compatible with a metastatic HCC with sarcomatous change. The symptoms ameliorated gradually after operation and he could walk with stick. Since the patient underwent radiotherapy before this operation, no further radiotherapy was arranged. However, bilateral lung metastases were diagnosed since July 23^rd^, 2009 during follow up.

**Figure 3:**
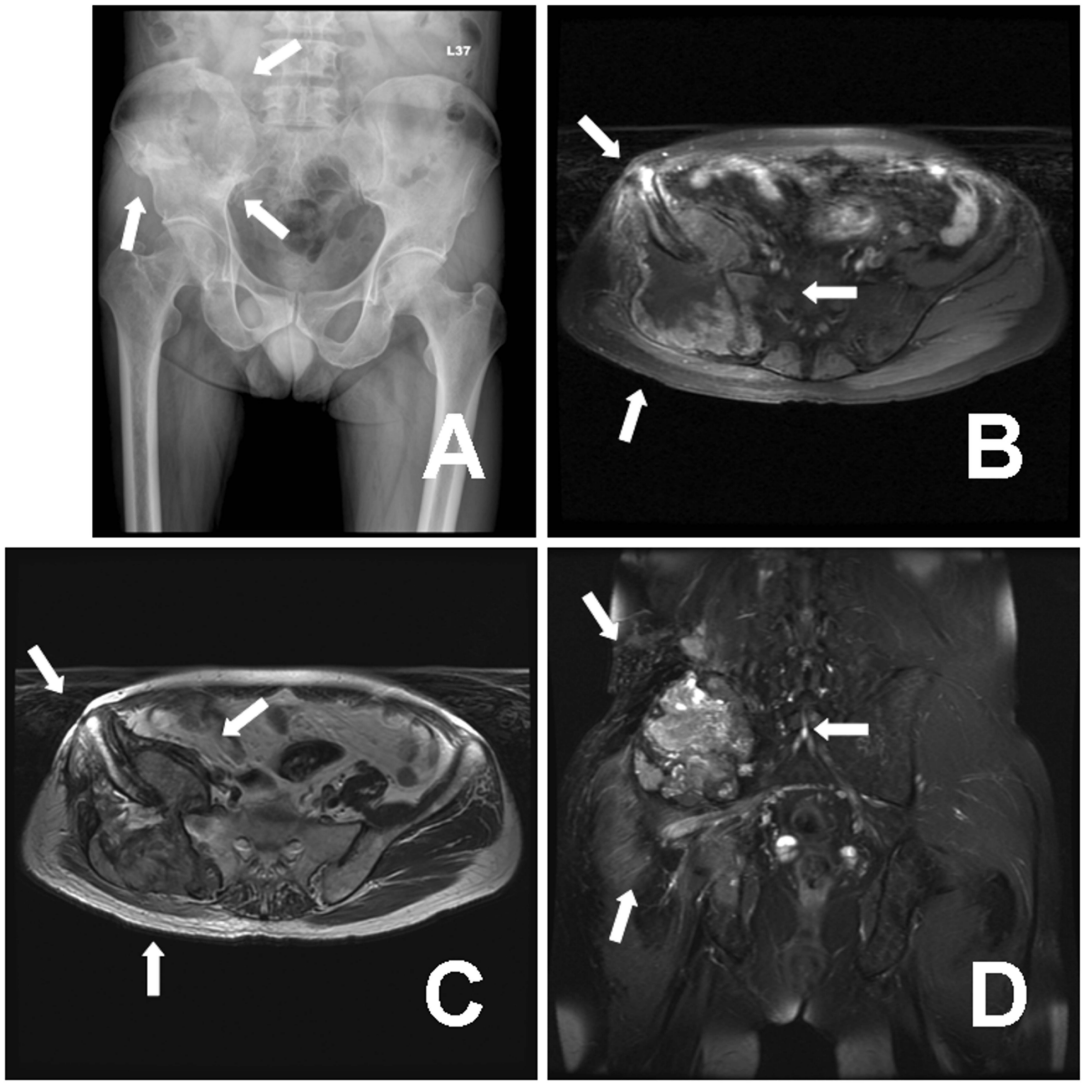
**Pelvis radiograph showed a large ill-defined osteolytic lesion at right iliac bone accompanied with iliac wing fracture (white arrows) (A)**. Repeated MRI revealed a 9.4 × 9.2 × 9.0 cm bipeduncular lesion arising from previous operation site, accompanied with iliac wing fracture (white arrows) (B to D). B: axial fast spin echo (FSE) fat-suppressed T1-weighted image with contrast enhancement. C: axial fast recover fast spin echo (frFSE) T2-weighted image. D: coronal fat-suppressed (FS) FSE T2-weighted image with contrast enhancement.

Unfortunately, another episode of right low back pain occurred since mid-September 2009. The pain was more severe compared with last time, especially when the patient ambulated. Follow up pelvic radiograph showed a large ill-defined osteolytic lesion at right iliac bone accompanied with iliac wing fracture (Fig 3-A; white arrows). Repeated MRI revealed a huge mass of 9.4 × 9.2 × 9.0 cm bipeduncular lesion arising from previous operation site, accompanied with iliac wing fracture (Fig 3-B to 3-D, white arrows). Tumor recurrence was impressed and the patient underwent tumor excision again on October 20^th^, 2009. Like previous operation, the tumor was very hypervascular with approximate 9 cm in diameter; iliac wing pathological fracture was also noted. The histopathological examination was compatible with earlier one. Compared with last report, the whole pictures were conclusive of progressive loss of hepatocytic carcinomatous characters and transitional to the differentiation of sarcomatous characters that included spindle cell, chondroid and osteoid components.

Since July 2008, serum AFP level was always within normal limit (< 20 ng/mL). Even with large bone lesions and systemic disease spreading, serum AFP level before each operation was only 13.92 and 15.39 ng/mL respectively.

## Discussion

Hepatocellular carcinoma with a spindle cell component has been referred to as sarcomatoid or sarcomatous HCC, spindle cell carcinoma, pseudosarcoma, and carcinosarcoma [3]. According to previous reports, 2% to 27% of HCC have a sarcomatous appearance, and the incidence is increasing with the use of more aggressive treatments [2,9].

The pathogenesis of the sarcomatous appearance of HCC has not yet been clarified because of a debate on whether it is derived from the transition of an ordinary HCC to a sarcomatous appearance or it is a double cancer of HCC and hepatic sarcoma [2,3,5,10]. Most investigators thought that the sarcomatous component is derived from a dedifferentiation of anaplasia from an ordinary HCC rather than double cancer [1,5]. Our pathologic analyses showed two patterns of tumor cells and the clinical course also suggested dedifferentiation of a metastatic HCC with sarcomatous change.

The typical findings of sarcomatoid HCC of liver is reported to be of massive expanding or multinodular confluent type with partial encapsulation [5]. Intrahepatic metastasis and adjacent organ invasion were relatively more common with sarcomatoid HCC than with ordinary HCC [11]. The metastatic lesions of our patient presented with bulky expansile dumbbell-shaped soft tissue masses, with relatively small bony destruction located at central portion; and often showed cold lesions on ^99m^Tc-MDP BS [4,6-8]. These characteristics suggested that the lesions originated from skeleton and gradually expanded as soft tumor masses almost without any bone related activity [4,6-8,12]. To our knowledge, only one previous report had metastatic HCC presented as sarcoma-like change with peritoneal dissemination [13]. Actually, the larger soft-tissue portion of the metastasis behaved more like a sarcoma.

Radiation induced sarcoma is a rare sequela of radiation therapy, and often occurred more than 10 years after radiotherapy [14,15]. The radiation dose in patients with radiation induced sarcoma is usually greater than 3,000 cGy [14]. Although we have no direct evidence that this sarcomatous change was associated with radiotherapy, but our patient received 5,250 cGy radiation and primary hepatic lesion revealed a typical HCC. These findings suggested the possibility of radiation induced sarcomatous transformation of HCC, although the interval between radiation and sarcomatous transformation in the case herein presented is a few months rather than the expected duration greater than 10 years.

On the other hand, serum AFP level never exceeded normal limit (< 20 ng/mL) in our patient since diagnosis of bone metastasis. Even with large tumor burden before both bone tumor excisions, the highest recorded serum AFP level was 15.39 ng/mL. This finding was compatible with the 11-patient series by Koo HR et al [5]. In their series, serum AFP level was more than 20 ng/mL in 7 patients (64%) and was more than 500 ng/mL in only 1 patient (9%).

In conclusion, we considered sarcomatoid HCC bone metastasis a highly aggressive lesion with unusual metastatic pattern. Surgical treatment with adequate safe margin in such a huge tumor with hypervascularity and extensive invasion in the pelvis was difficult; and radiotherapy maybe refractory regarding the sarcomatous nature. Therefore, debulking operation with local symptoms control may provide a better quality of life. ^99m^Tc-MDP BS often has false-negative results and serum AFP level is not an appropriate marker for monitoring of bone metastasis of sarcomatoid HCC. Besides, the clinical course suggests sarcomatoid HCC is derived from the transition of an ordinary HCC.

## Competing interests

The authors declare that they have no competing interests.

## Authors' contributions

CYC wrote the manuscript and designed the report. YTH revised the manuscript. TYL collected the patient data and pathologic pictures. WHL participated in pathologic analysis. KW did the pathologic consultation. RSY conceived of the study and participated in the design and coordination. All authors read and approved the final manuscript.

## Consent

Written informed consent was obtained from the patient for publication of this case report and accompanying images. A copy of the written consent is available for review by the Editor-in-Chief of this journal.
